# Separate signaling events control TCR downregulation and T cell activation in primary human T cells

**DOI:** 10.1002/iid3.383

**Published:** 2020-12-22

**Authors:** Lieve E. H. van der Donk, Louis S. Ates, Jet van der Spek, Laura M. Tukker, Teunis B. H. Geijtenbeek, Jeroen W. J. van Heijst

**Affiliations:** ^1^ Department of Experimental Immunology, Amsterdam Infection and Immunity Institute, Amsterdam UMC University of Amsterdam Amsterdam The Netherlands; ^2^ Neogene Therapeutics Amsterdam The Netherlands

**Keywords:** CD4^+^ T cells, protein tyrosine kinases, T‐cell activation | T‐cell receptor, TCR downregulation

## Abstract

**Introduction:**

T‐cell antigen receptor (TCR) interaction with cognate peptide:MHC complexes trigger clustering of TCR:CD3 complexes and signal transduction. Triggered TCR:CD3 complexes are rapidly internalized and degraded in a process called ligand‐induced TCR downregulation. Classic studies in immortalized T‐cell lines have revealed a major role for the Src family kinase Lck in TCR downregulation. However, to what extent a similar mechanism operates in primary human T cells remains unclear.

**Methods:**

Here, we developed an anti‐CD3‐mediated TCR downregulation assay, in which T‐cell gene expression in primary human T cells can be knocked down by microRNA constructs. In parallel, we used CRISPR/Cas9‐mediated knockout in Jurkat cells for validation experiments.

**Results:**

We efficiently knocked down the expression of tyrosine kinases Lck, Fyn, and ZAP70, and found that, whereas this impaired T cell activation and effector function, TCR downregulation was not affected. Although TCR downregulation was marginally inhibited by the simultaneous knockdown of Lck and Fyn, its full abrogation required broad‐acting tyrosine kinase inhibitors.

**Conclusions:**

These data suggest that there is substantial redundancy in the contribution of individual tyrosine kinases to TCR downregulation in primary human T cells. Our results highlight that TCR downregulation and T cell activation are controlled by different signaling events and illustrate the need for further research to untangle these processes.

## Graphical abstract

T‐cell antigen receptor (TCR) interaction with cognate peptide:MHC complexes triggers internalization and degradation of the TCR. To elucidate which molecular players are involved, we knocked down Src family kinases Lck and Fyn, and the kinase ZAP70. We found that though T cell activation and effector function were impaired, TCR downregulation was unaffected, suggesting that TCR downregulation and T cell activation are controlled by different signaling events.

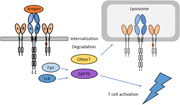

## 1 INTRODUCTION

T cells are essential players in the adaptive immune responses that are needed to protect against infections and cancer. T‐cell receptors (TCRs) on T cells recognize peptide antigens presented on major histocompatibility complex (MHC) by antigen‐presenting cells (APCs). This specific recognition, bolstered by costimulatory molecule interactions, induces signaling pathways that lead to T‐cell differentiation, effector functions, and survival.^1^

The TCR is comprised of a genetically diverse α and β chain that together confer antigen specificity. To form the fully functional TCR, TCRα/β are noncovalently associated with the invariant CD3γε and CD3δε subunits and the TCRζζ homodimer.^2^ The cytoplasmic domains of CD3ε, CD3δ, CD3γ, and TCRζ all contain immunoreceptor tyrosine‐based activation motifs (ITAMs).^3^ After the TCR is triggered through recognition of peptide:MHC, intracellular signaling is initiated by phosphorylation of these ITAMs by Src family kinases, of which Lck and Fyn are most abundantly expressed throughout the lifespan of human T cells.^4^, ^5^ Lymphocyte‐specific protein tyrosine kinase (Lck) is the dominant kinase initiating T cell activation through ITAM phosphorylation.^6^, ^7^, ^8^ Lck is non‐covalently attached to the coreceptors CD4 and CD8^9^, ^10^ and is thereby recruited to the TCR after recognition of a peptide:MHC complex. Phosphorylated ITAMs serve as docking sites for the Syk family tyrosine kinase ZAP70, which is activated through phosphorylation by Lck.^11^, ^12^ ZAP70, in turn, phosphorylates downstream molecules, such as LAT^13^ and SLP76,^14^ which eventually leads to T cell activation.^1^, ^7^, ^8^, ^15^

T cell activation is tightly regulated to induce specific responses, while preventing hyperreactivity that could lead to damage to healthy tissues and autoimmunity. One such T cell regulation mechanism is the rapid internalization and active degradation of the TCR upon TCR triggering, a process that is called ligand‐induced TCR downregulation.^16^, ^17^, ^18^, ^19^ The extent of TCR downregulation is generally proportional to the strength of signaling input, meaning that higher affinity ligands induce greater TCR downregulation.^20^, ^21^ In addition, we recently observed that clonally‐expanded T cells also display persistent TCR downregulation, the extent of which is programmed by the strength of the initial T‐cell antigen recognition.^22^ Such T cells with adjusted TCR expression display an increased threshold for cytokine production and renewed proliferation upon secondary antigen encounter, and, therefore, presumably are better equipped to execute a balanced immune response. These findings underscore that downregulation of the TCR is an important protective mechanism against hyperreactive T‐cell responses that could harm the host.^23^, ^24^ Understanding the molecular mechanisms that underlie TCR downregulation is, therefore, important to fine‐tune immunotherapeutic approaches, either by preventing TCR downregulation in, for example, CAR‐T‐cell therapies, or by inducing it in patients with autoimmune disorders.

Because TCR triggering and TCR downregulation are tightly linked,^1^, ^25^, ^26^ it is possible that the upstream molecular pathways of T cell activation and TCR downregulation are similar. Besides the importance of Lck for T‐cell activation, it has also been described to be involved in TCR downregulation. Specifically, Lck was described to control TCR downregulation through phosphorylation of ITAMs in CD3 and TCRζ.^27^ Chemical inactivation of Lck in immortalized Jurkat T cells inhibited TCR downregulation,^16^, ^28^ and, conversely, a constitutively active form of Lck caused rapid internalization of cell surface TCR:CD3 complexes and their degradation in lysosomes.^16^ Although the molecular pathways of TCR downregulation have been studied in detail, the majority of these findings have been obtained in mouse models and immortalized T‐cell lines, instead of human primary T cells.^7^, ^8^, ^11^, ^15^, ^16^, ^29^, ^30^, ^31^ Importantly, cellular processes such as the dynamics of TCR internalization may differ markedly between primary T cells and Jurkat cells, and between mice and man. For instance, TCR recycling in T‐cell hybridoma cells is reported to be faster than in naïve CD4^+^ T cells^17^ and the constitutive degradation rate of TCRζ and CD3ε is slower in primary cells than in Jurkat cells.^32^ Furthermore, human primary T cells and Jurkat cells have distinctive patterns of cytokine release and coreceptor expression, and they induce different phosphorylation levels of target molecules.^33^ Because of these fundamental differences between human primary T cells and Jurkat cells, we set out to investigate the molecular mechanisms of TCR downregulation in a more physiological setting and developed a model to study this by efficient gene knockdown in human primary T cells.

Our data strongly suggest that Src family kinases as a group are required for TCR downregulation in human primary T cells. However, its members Lck and Fyn as well as the directly downstream kinase ZAP70 are individually redundant in this process, despite having profound impact on T cell activation and effector functions. Thus, this study highlights that TCR downregulation and T cell activation are separable molecular processes and provides the tools to further unravel these pathways in primary human cells.

## 2 MATERIALS AND METHODS

### 2.1 Cell lines

Human embryonic kidney cells that contain the mutant version of the SV40 large T‐cell antigen (HEK293T cells) were used to generate lentiviruses. Platinum A (PLAT‐A) cells are retroviral packaging cells, used to generate retroviruses.^34^ Both the HEK293T and PLAT‐A cell lines were maintained in IMDM (Gibco) containing 10% fetal bovine serum (FBS) (Sigma‐Aldrich) and 10,000 U/ml penicillin/streptomycin. Jurkat cells are a human acute leukemic T‐cell line, which was maintained in Rosewell park Memorial Institute (RPMI) 1640 medium (Gibco) with l‐glutamine, 50 μM β‐mercaptoethanol, 10% FBS, and 10,000 U/ml penicillin/streptomycin (complete RPMI).

### 2.2 Primary cells

This study was performed according to the Amsterdam University Medical Centers, location AMC Medical Ethics Committee guidelines and all donors gave written informed consent in accordance with the Declaration of Helsinki. Human peripheral blood lymphocytes (PBLs) were isolated from buffy coats by density gradient centrifugation on Lymphoprep (Nycomed) and Percoll (Pharmacia). PBLs were activated in non‐tissue culture‐treated six‐well plates (Greiner Bio‐One) coated with 2.5‐μg/ml plate‐bound anti‐CD3 (UCHT‐1) and anti‐CD28 (CD28.2; both Biolegend) diluted in 0.1 M sodium bicarbonate buffer, and cultured in complete RPMI supplemented with 10 ng/ml interleukin‐2 (IL‐2) (Preprotech). After activation, the PBLs were maintained in complete RPMI supplemented with IL‐2.

### 2.3 Construction of retroviral and lentiviral microRNA vectors and lentiviral CRISPR vectors

For retroviral transductions, five to six microRNA oligonucleotide sequences per gene were selected from the Genetic Perturbation Platform (Broad Institute) and ordered from Sigma‐Aldrich. A microRNA sequence targeting luciferase (RLuc) was used as control. Using standard molecular cloning techniques, the microRNA was cloned into a modified pMY backbone (Cell Biolabs) containing the marker Ly6G or CD90.2 (van der Donk et al. In preparation). To enable lentiviral microRNA transductions, the marker and microRNA cassette were cloned into a modified lentiCRISPR v2 backbone (Addgene #52961; kind gift from Prof. Dr. N. Zelcer). For lentiviral CRISPR/Cas9 transductions, four CRISPR guideRNAs were selected per gene from the Toronto human knockout pooled library (TKOv3),^35^ ordered from Sigma‐Aldrich, annealed, and cloned into the lentiCRISPR v2 backbone using standard molecular cloning techniques. A guideRNA targeting hAAVS1 was used as control.

### 2.4 Retroviral transfection of PLAT‐A and lentiviral transfection of HEK293T, and transduction of T cells

For retroviral transfection of PLAT‐A cells,^34^ 2.5 × 10^6^ cells per condition were plated in Advanced TC six‐well plates (Greiner Bio‐One), coated with poly‐d‐lysine (Sigma‐Aldrich). The cells were incubated overnight at 37°C in the presence of transfection complexes containing 2 μg pMY vector, 0.4 μg pCL‐Ampho (Novus Biologicals^36^), 0.4 μg DGCR8 siRNA (Qiagen^37^), P3000 and Lipofectamine 3000 (Thermofisher), supplemented with Opti‐MEM (Gibco). For lentiviral transfection of HEK293T cells, 2.5 × 10^6^ cells were used per condition in a regular six‐well plate. The cells were incubated overnight at 37°C in the presence of transfection complexes containing 1 μg lentiviral vector, 0.6 μg pMDLg/pRRE (Addgene #12251), 0.2 μg pRSV‐Rev (Addgene #12253), 0.3 μg pMD2.G (Addgene #12259),^38^ 0.4 μg DGCR8 siRNA (not required for lentiviral CRISPR experiments), P3000 and Lipofectamine 3000. After overnight incubation, the supernatants of transfected cells were replaced with 1.5 ml complete RPMI. Forty‐eight hours after transfection, viral supernatants were harvested, filtered over a 0.45‐μm filter, and used to transduce T cells. The remaining HEK293T or PLAT‐A cells were washed with phosphate‐buffered saline (PBS) and resuspended for fluorescence‐activated cell sorting (FACS) analysis to determine the transfection efficiency.

Forty‐eight hours before transduction, PBLs were stimulated at 37°C on non‐tissue culture‐treated six‐well plates that were coated with 2.5 μg/ml anti‐human CD3 (UCHT1) and anti‐human CD28 (CD28.2). For the transduction, 1 × 10^6^ Jurkat cells or PBLs were plated on retronectin‐coated plates (2.5 μg/ml; Takara), before adding 1 ml of virus‐containing supernatant. The cells were then centrifuged for 2 h at 1000*g* at 32°C, followed by 3 h incubation at 37°C, after which 2 ml fresh complete RPMI was added per well (for PBLs containing IL‐2). At 2 days (before antibiotic selection) and 5 days (after antibiotic selection) after transduction, cells were harvested and stained for flow cytometry as described below. Antibiotic selection during 72 h was performed by adding 5 μg/ml puromycin or 10 μg/ml blasticidin, depending on the vector.

### 2.5 Flow cytometry staining

All cells were first stained for viability using Fixable Viability Dye eFluor™ 780 (1:1000) (eBioscience). For cell surface staining, cells were incubated in FACS buffer (PBS containing 0.5% bovine serum albumin [BSA; Sigma‐Aldrich] and 0.1% NaN_3_) containing antibodies for 10 min at 4°C. Then, cells were fixed with 2% paraformaldehyde (PFA; Electron Microscopy Sciences) for 5 min at 4°C. For intracellular staining, the fixation step was followed by a permeabilization step in Perm/Wash solution (BD Biosciences) for 5 min at 4°C, and an intracellular staining step with antibodies diluted in Perm/Wash solution for 10 min at 4°C. The antibody clones and manufacturers used are listed in Table S1. Single‐cell measurements were performed on a FACS Canto flow cytometer (BD Biosciences) and FlowJo V10 software (TreeStar) was used to analyze the data. For each flow cytometry experiment, viable single cells were gated, after which Jurkat cells were selected on CD45 expression, and PBLs were selected on CD8 or CD4 expression. For transduction experiments, the Jurkat cells were gated on FLAG, and PBLs on Ly6G or CD90.2 (or both) and then on CD4. Exceptionally, the Ly6G^+^ Lck‐KD, and Ly6G^+^CD90.2^+^ Lck/Fyn‐KD PBLs were not gated on CD4, but the total T cells were assessed.

### 2.6 Fyn antibody conjugation

As there is no commercial flow cytometry antibody available for Fyn, we conjugated our immunoblot antibody for Fyn (Table S1) to a fluorochrome with a Lightning‐Link® conjugation kit (Expedeon) according to the manufacturer's instructions.

### 2.7 Optimization of T cell stimulations: Testing the steric hindrance of antibodies using a primary and secondary staining

To investigate steric hindrance of antibodies, 1 × 10^5^ Jurkat cells or PBLs were seeded per well in tissue‐treated 96‐well plates (Greiner Bio‐One). Cells were stained in several rounds to test the accessibility of the target protein for the secondary, fluorochrome‐labeled antibody after staining with a primary antibody. First, cells were stained with FACS buffer, or 2.5 μg/ml of various anti‐CD3 antibodies (purified UCHT1, purified OKT3, purified SK7, purified HIT3a, or fluorescein isothiocyanate [FITC]‐labeled UCHT1) for 10 min at 4°C. After washing, the cells were stained with an antibody solution to assess different surface markers. For Jurkat, the secondary antibody solution contained 5 μg/ml fluorochrome‐labeled anti‐CD45 and anti‐TCRβ. For PBLs, the secondary antibody solution contained 5 μg/ml fluorochrome‐labeled anti‐CD4, anti‐CD8α, anti‐CD27, anti‐CD45RA, and anti‐TCRβ. Cells were fixed with 2% PFA, followed by intracellular staining with anti‐TCRζ, and analyzed with flow cytometry. The percentage of hindrance by each antibody was determined from the mean fluorescence intensity (MFI), using the buffer control as reference, such that the expression of an indicated molecule in the buffer control was set at 100% (e.g., Expression % TCRβ = [MFI condition 1/MFI buffer control] × 100%). Cells were stimulated with the anti‐CD3ε clone UCHT1, unless indicated otherwise.

### 2.8 TCR downregulation assay

A TCR downregulation assay was set up and performed to determine the extent of TCR expression of stimulated samples versus unstimulated controls. A non‐tissue culture‐treated 96‐well plate was coated overnight at 4°C with either an isotype control (antimouse immunoglobulin G [IgG] [−]) or anti‐human CD3 (low dose 0.25 μg/ml [+]; high dose 2.5 μg/ml [++]) and anti‐human CD28, diluted in 0.1 M sodium bicarbonate buffer. Samples are stimulated with the high dose, unless indicated otherwise. Plates were blocked with 2% BSA in PBS and washed once with PBS. T cells (1 × 10^5^) were seeded per well and incubated for the indicated duration at 37°C. After incubation ice‐cold MACS (magnetic‐activated cell sorting) buffer (PBS supplemented with 0.5% BSA and 2 mM EDTA) was added and cells were transferred to a noncoated 96‐well plate. The cells were stained for flow cytometry as described above, including antibodies targeting TCRβ, TCRζ, and CD69.

This assay was performed in the presence of various inhibitors. The cells were preincubated in the presence of 20 μM PP2 (Sigma‐Aldrich), 100 nM Dasatinib (Sigma‐Aldrich), 50 μM Imatinib Mesylate (Selleckchem), or 1 μM Bafilomycin A1 (Invivogen) for 1 h at 37°C before directly being transferred to the antibody‐coated plate. Stimulation and staining is similar as described above.

### 2.9 Immunoblot analysis

After antibiotic selection, the cells were spun down and resuspended in radioimmunoprecipitation assay buffer (Cell Signaling) supplemented with protease and phosphatase inhibitors (Roche). Then, five‐times concentrated Laemmli sample buffer was added and the lysates were incubated for 10 min at 95°C before separation by sodium dodecyl sulfate‐polyacrylamide gel electrophoresis. Proteins were transferred to nitrocellulose membranes (GE Healthcare Life Science) or Immobilon®‐FL PVDF membranes (Sigma‐Aldrich) by immunoblot and stained with Ponceau red (Sigma‐Aldrich), followed by blocking in 5% milk (Sigma‐Aldrich). Blots were incubated O/N at 4°C with indicated primary antibodies diluted in 1% milk in Tris‐buffered saline with Tween‐20 (TBST): Anti‐β‐actin and anti‐Fyn. Secondary antibodies, diluted in 1% milk, were IRDye 800CW goat‐anti‐mouse IgG (H+L) and IRDye 680RD goat‐anti‐rat IgG (H+L) (Li‐Cor). Measurements were performed on the Odyssey, and analyzed with Odyssey V3.0 software (Li‐Cor Biosciences).

To assess total phosphorylation by immunoblot, 5 × 10^6^ Jurkat cells were stimulated with either anti‐mouse IgG1 or anti‐mouse IgG1 and anti‐human CD3 (UCHT1). The cells were stimulated for 2 or 5 min at 37°C, followed by direct addition of 10×‐excess ice‐cold FACS buffer. Cells were lysed and blotted as described above, except that blots were blocked in 5% BSA in PBS, and the primary (anti‐mouse/human β‐actin, anti‐phosphorylated tyrosine) and secondary antibodies were diluted in 1% BSA/TBST.

### 2.10 Cytokine production assay

A non‐tissue culture‐treated 96‐well plate was coated with 0.1 M sodium bicarbonate buffer or 2.5 μg/ml anti‐human CD3 and 2.5 μg/ml anti‐human CD28 diluted in buffer, and incubated overnight at 4°C. Plates were blocked with 2% BSA in PBS, and washed once with PBS. T cells (2 × 10^5^) were seeded per well and resuspended in either RPMI with Brefeldin‐A (eBioscience; unstimulated control and antibody stimulated samples) or with Brefeldin‐A, PMA (50 ng/ml, Sigma‐Aldrich) and ionomycin (1 μg/ml, Sigma‐Aldrich) (positive control) and incubated for 5 h at 37°C. Cells were stained for flow cytometry as described above.

### 2.11 Statistical analysis

**Figure 1:**
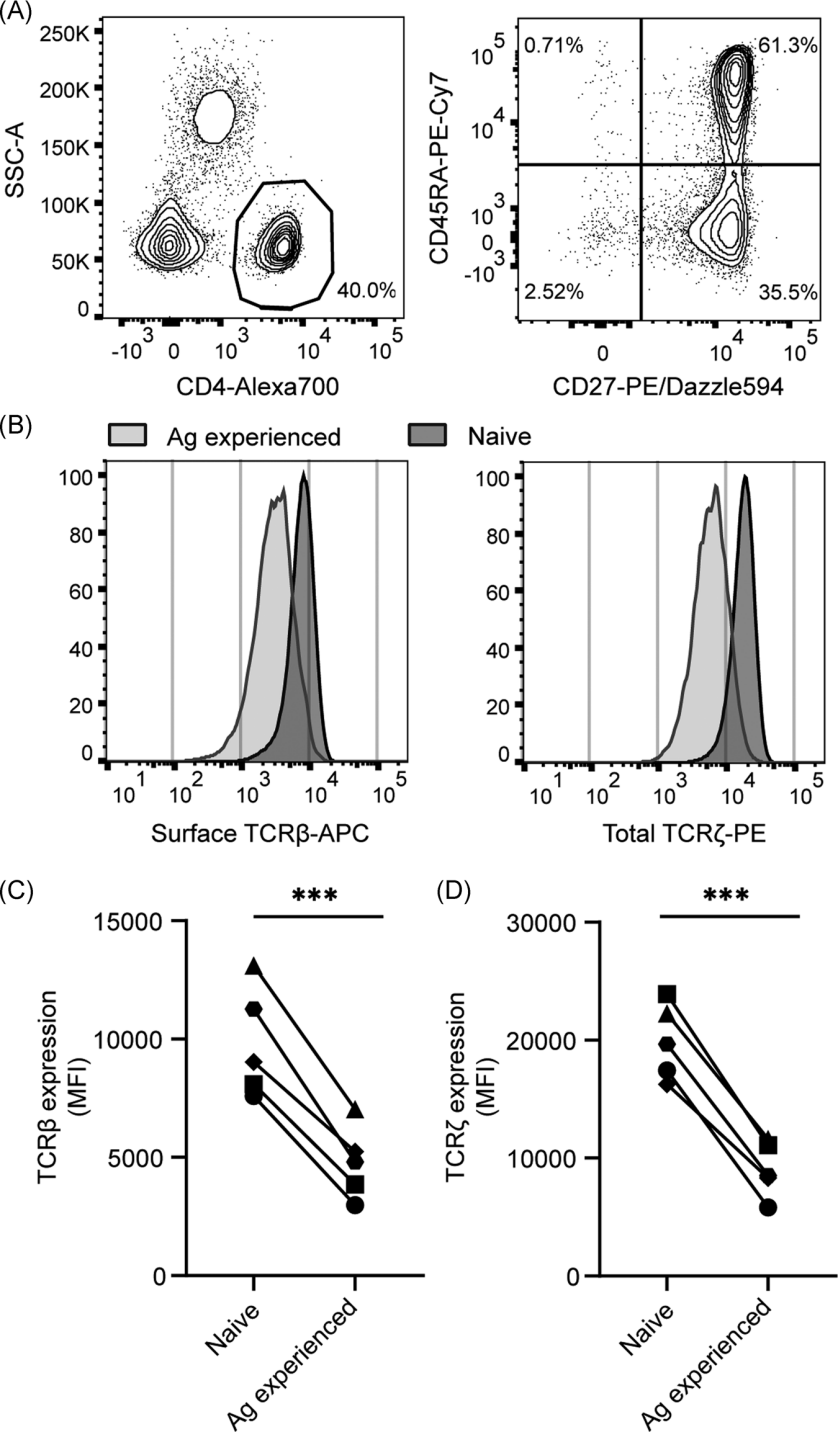
Reduced TCR expression in antigen‐experienced primary human CD4^+ ^T cells. Human PBLs from healthy donors were gated (A) to select CD4^+^, CD45RA^+^CD27^+^, naive and CD45RA^−^CD27^−^ antigen‐experienced T cells and measure surface TCRβ and total TCRζ expression within these separate populations (B). Data in (A) and (B) are one representative experiment of the five biological replicates depicted in (C) and (D). (C,D) Aggregate MFI data for surface TCRβ (C) and total TCRζ (D) expression for *n* = 5 donors in one experiment, with each symbol representing an individual donor. MFI, mean fluorescence intensity; PBL, peripheral blood lymphocytes; TCR, T‐cell receptor. Paired Student's *t* test, ***p < .001

**Figure 2:**
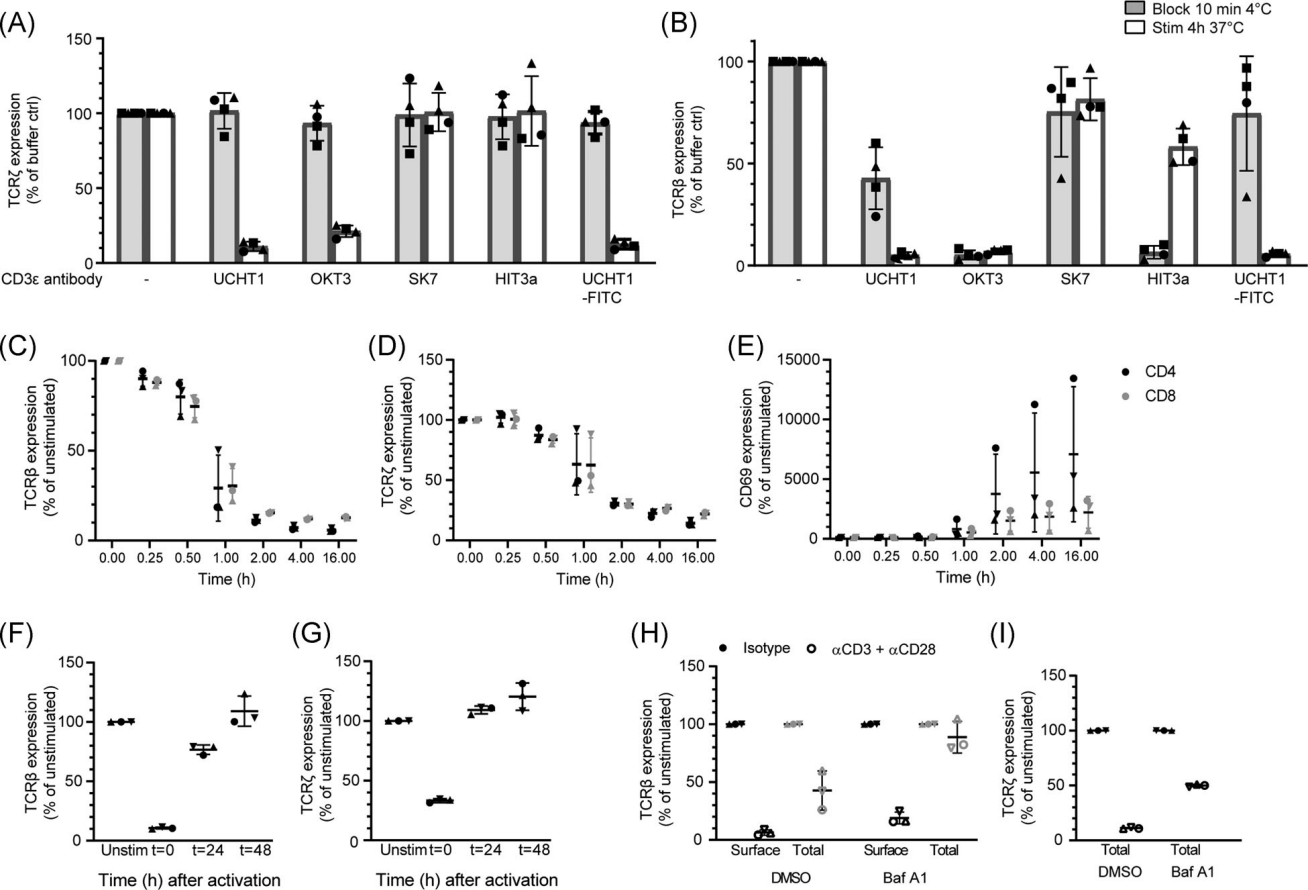
TCR downregulation includes internalization and lysosomal degradation. (A) Total relative TCRζ expression in human CD4^+^ T cells after stimulation with different anti‐CD3ε clones. SK7 and HIT3a did not induce TCR downregulation. (B) Surface TCRβ staining of human CD4^+^ T cells revealed that UCHT1 induced TCR downregulation, without causing steric hindrance. (C–G) Kinetics of relative surface TCRβ (C) and total TCRζ (D) expression, CD69 upregulation (E) in human CD4^+^ and CD8^+^ T cells from healthy donors (*n* = 3) after stimulation with anti‐CD3 (UCHT1)/CD28. Relative surface TCRβ (F) and total TCRζ (G) expression before, during, and after TCR triggering of human CD4^+^ T cells. (H, I) Relative surface (black) and total (grey) TCRβ (H) and total TCRζ (I) expression of human CD4^+^PBLs after 4h stimulation with anti‐CD3/CD28 in the presence of Bafilomycin A1. (C–I) Data are depicted as normalized MFI. Symbols represent individual donors (*n* = 3) with bars depicting the median and 95% CI. (A, B) Data are from two independent experiments with in total *n* = 3 individual donors. (C–I) Data from one experiment with *n* = 3 individual donors. CI, confidence interval; MFI, mean fluorescence intensity; PBL, peripheral blood lymphocytes; TCR, T‐cell receptor

Graphpad Prism v8 (GraphPad Software) was used to generate all graphs and for statistical analyses. Statistics were performed using a Student's *t* test for pairwise comparisons (Figures 1, 4–6). Multiple comparisons within groups were performed using an RM one‐way analysis of variance with a Tukey's multiple comparisons test (Figure 2). *p* < .05 were considered statistically significant.

## 3 RESULTS

### 3.1 Antigen‐experienced human T cells display lower TCR expression levels

First, we investigated whether we could detect evidence of persistent TCR downregulation in human T cells, similar to what has been described recently in animal models.^22^ To this end, we isolated peripheral blood lymphocytes (PBLs) from five healthy donors and compared TCRβ surface levels on antigen‐experienced (CD45RA^−^ CD27^−^) with those on naïve (CD45RA^+^ CD27^+^) CD4^+^ T cells (Figure 1A,B). Surface TCRβ levels were significantly reduced on antigen‐experienced compared to naïve T cells (Figure 1C; *p* < .001). Similarly, antigen‐experienced T cells had lower levels of intracellular TCRζ compared to naïve T cells (Figure 1D; *p* < .001). These data strongly suggest that human antigen‐experienced CD4^+^ T cells have lower TCR expression compared to their naïve counterparts. Therefore, TCR downregulation appears to form an integral part of human T‐cell responses.

### 3.2 CD3/CD28 crosslinking induces TCR downregulation in primary human T cells

**Figure 3:**
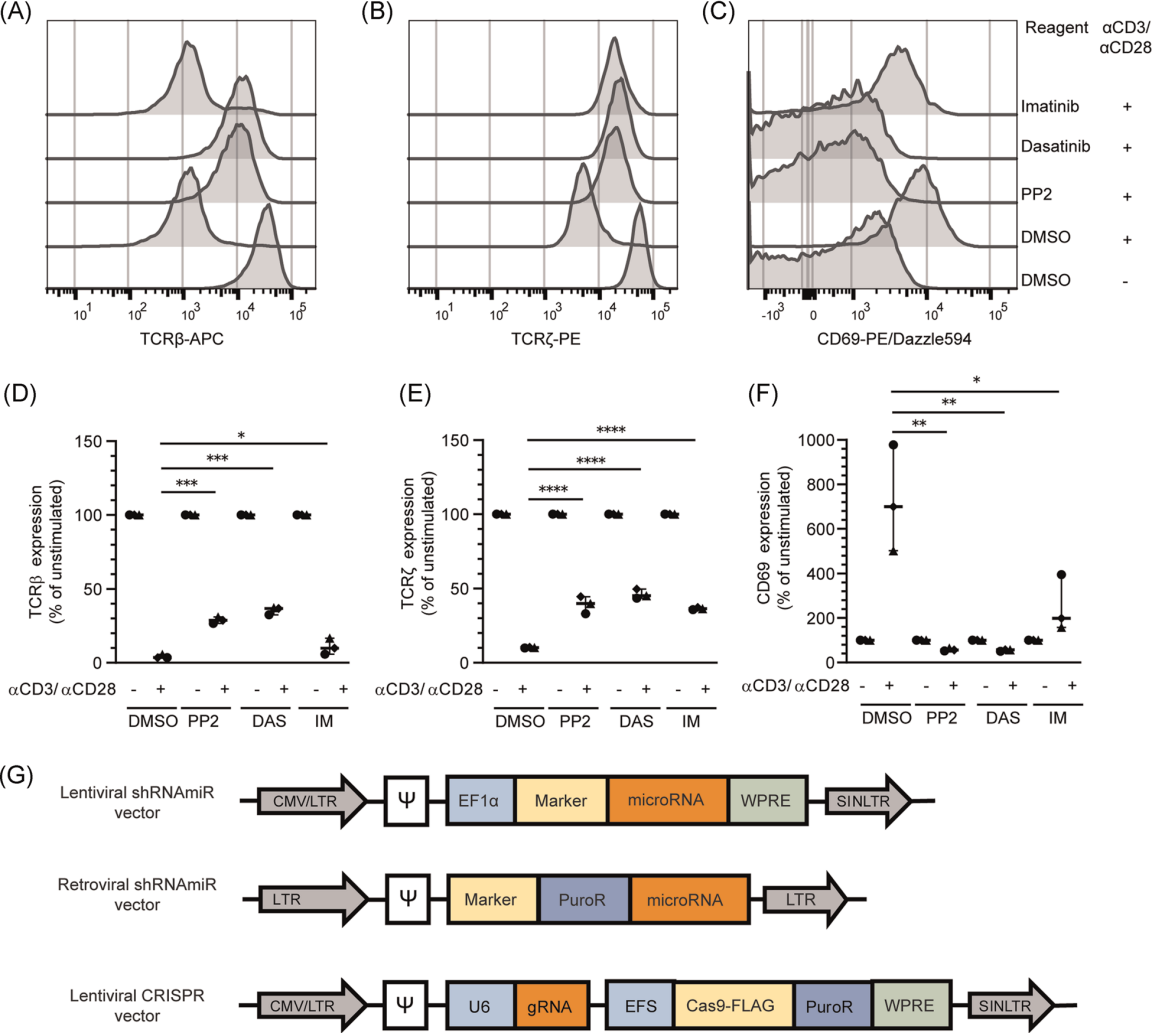
Chemical inhibitors suggest that TCR downregulation is dependent on multiple tyrosine kinases. Human CD4^+^PBLs were stimulated with anti‐CD3/CD28 in the presence of protein tyrosine kinase inhibitors PP2, dasatinib, or imatinib. (A–C) Representative histograms of surface TCRβ (A), total TCRζ (B) expression, and surface CD69 (C) expression. (D–F) Aggregate data normalized to the unstimulated control for *n* = 3 individual donors examined in a single experiment, with the median and 95% CI depicted. (G) Lentiviral and retroviral microRNA vectors and lentiviral CRISPR/Cas9 vectors for knockdown of TCRζ (*CD247*) expression. CI, confidence interval; MFI, mean fluorescence intensity; PBL, peripheral blood lymphocytes; TCR, T‐cell receptor. Statistics were calculated by one‐way analysis of variance followed by Tukey's test for multiple comparisons, *p < .05; **p < .005; ***p < .001; ****p < .0001

To investigate the mechanisms underlying TCR downregulation in PBLs, we optimized an antigen‐independent TCR downregulation assay using plate‐bound antibodies against CD3ε and CD28.^17^, ^25^, ^39^ Though T cell stimulation with anti‐CD3ε/CD28 antibodies is a well‐known and effective method to study ligand‐induced TCR downregulation, it is important to consider that the CD3ε and TCRβ subunits are present in close proximity on the T‐cell surface. This implies that anti‐CD3ε antibodies used to stimulate T cells, could sterically hinder the binding of fluorochrome‐labeled anti‐TCRβ antibodies used for the flow cytometry‐based readout. In previous studies, such a potential blocking effect of anti‐CD3 antibodies was not investigated.^31^, ^32^, ^40^ To optimize the T cell stimulation for PBLs and Jurkat cells, we investigated the steric hindrance of five different anti‐CD3ε antibodies, by staining PBLs (Figure S1A,B) and Jurkat cells (Figure S1C,D) at 4°C. Detection of surface TCRβ by flow cytometry was impaired after staining with HIT3a and OKT3, suggesting steric hindrance. The capacity to induce TCR downregulation in PBLs (Figure S1E,F) and Jurkat cells (Figure S1G,H) was assessed by stimulation with the different plate‐bound anti‐CD3ε antibodies. Surface TCRβ and total TCRζ expression were both reduced in response to stimulation with UCHT1 in PBLs as well as Jurkat cells. Interestingly, FITC‐labeled UCHT1 induced the lowest level of steric hindrance of all antibodies in both cell types (Figures 3A,B, and S1I,J). Thus, UCHT1 induced minimal steric hindrance, while efficiently inducing TCR downregulation and was, therefore, selected for all subsequent assays.

### 3.3 Transient downregulation of TCR complexes via lysosomal degradation

With the optimized assay, preactivated PBLs were stimulated and the kinetics of ligand‐induced TCR downregulation as well as CD69 upregulation as a marker of T cell activation were determined. Surface TCRβ and total TCRζ expression was quickly downregulated after stimulation, with similar kinetics for CD4^+^ and CD8^+^ T cells (Figure 3C,D). TCR downregulation was detected as early as 15 min after stimulation and reached its maximum after 2–4 h, which is in accordance with previous literature on human T‐cell clones.^41^ CD69 upregulation was consistently detected between donors and increased further until 16 h after activation, which was, therefore, selected as optimal timepoint in this assay (Figure 3E). During continued anti‐CD3 stimulation, surface TCRβ and total TCRζ expression remained low, but expression recovered after removal of the stimuli and returned to baseline after 48 h (Figure 3F,G).

It is currently thought that the TCR:CD3 complex is degraded in lysosomes after it is internalized upon T cell stimulation.^16^, ^41^ We, therefore, analyzed the expression of surface as well as total levels of both TCRβ and TCRζ in preactivated PBLs following anti‐CD3/CD28 stimulation using the lysosomal acidification inhibitor Bafilomycin A1 (Figure 3H,I). TCRβ‐degradation was completely blocked by Bafilomycin A1, while surface downregulation still occurred. Moreover, approximately two‐fold higher levels of TCRζ remained in cells pretreated with Bafilomycin A1. Thus, these data confirm that triggered TCR:CD3 complexes are internalized and subsequently degraded in lysosomes.

### 3.4 TCR downregulation is dependent on tyrosine kinases

The first proteins downstream of TCR triggering and signaling are the protein tyrosine kinases (PTKs) Lck and Fyn.^1^, ^5^, ^42^ To investigate the role of these PTKs in TCR downregulation of primary human T cells, preactivated PBLs from healthy donors were stimulated with anti‐CD3/CD28 in the presence of different tyrosine kinase inhibitors. PP2 and dasatinib are tyrosine kinase inhibitors with a broad range of targets in the Src kinase family.^43^, ^44^ In contrast, though imatinib is a well‐known Abl inhibitor, it is also described as a more specific inhibitor for Lck in T cells.^44^, ^45^ PP2 and dasatinib strongly inhibited TCR downregulation (Figures 2A,B, and 2D,E). In addition, T cell activation was fully blocked as the inhibitors prevented CD69 upregulation (Figures 2C and 2F). Strikingly, imatinib inhibited TCRζ degradation just as efficiently as PP2 and dasatinib, but did not inhibit TCRβ downregulation and only partially inhibited CD69 upregulation. These data suggest that inhibition by imatinib is either weaker, or more specific, leading to an intermediate phenotype, whereas broad‐acting inhibitors PP2 or dasatinib blocked complete TCR downregulation. Thus, these data suggest that a certain phosphorylation threshold is required, or that a combination of Src‐family kinases, or another (yet unknown) kinase besides Lck is involved, which is inhibited by PP2 and dasatinib, but not by imatinib.^44^, ^45^

### 3.5 Genetic modification to investigate TCR downregulation in T cells

Next, we more precisely investigated the role of the individual kinases in TCR downregulation using different genetic perturbation methods. To select the optimal method for genetic perturbation in our T‐cell models, we generated lentiviral and retroviral vectors coding for microRNAs and lentiviral CRISPR/Cas9 vectors to knock‐down or knock‐out PTK genes, respectively. First, we compared the transduction and genetic perturbation efficiency of each vector in PBLs (Figure 2G). After transduction of PBLs with the lentiviral CRISPR/Cas9 vectors, only a minimal level of Cas9‐FLAG‐staining was detected and no viable cells remained after antibiotic selection (data not shown). These data confirm reports by others^46^, ^47^ that such lentiviral CRISPR vectors are not a viable tool to achieve gene knockouts in primary T cells. As an alternative method for PBLs, we compared lentiviral and retroviral microRNA knockdown. For proof of principle, we used different vectors containing microRNAs that target TCRζ (*CD247*) with different knockdown efficiencies (Figure S1K,L). The lentiviral microRNA vectors transduced PBLs very efficiently, as shown by a high percentage of Ly6G positive cells (Figure S1M). However, within the transduced population (Ly6G^+^) it appears that the retroviral vectors induced better knockdown of TCRζ (Figure S1N) and further reduced surface expression of TCRβ than the lentiviral vectors (Figure S10).^1^, ^48^ In contrast to the PBLs, the lentiviral CRISPR/Cas9 vectors were efficiently transduced into Jurkat cells, and the proportion of viable cells remained high after antibiotic selection. Within the FLAG^+^ population, efficient knockout of TCRζ was achieved, and subsequent reduction of surface TCRβ was observed (Figure S1P). Therefore, we used CRISPR‐mediated gene knockout as the standard method for Jurkat cells and retroviral microRNA‐based gene knockdown for primary T cells.

### 3.6 Lck and Fyn are individually redundant for ligand‐induced TCR downregulation

**Figure 4:**
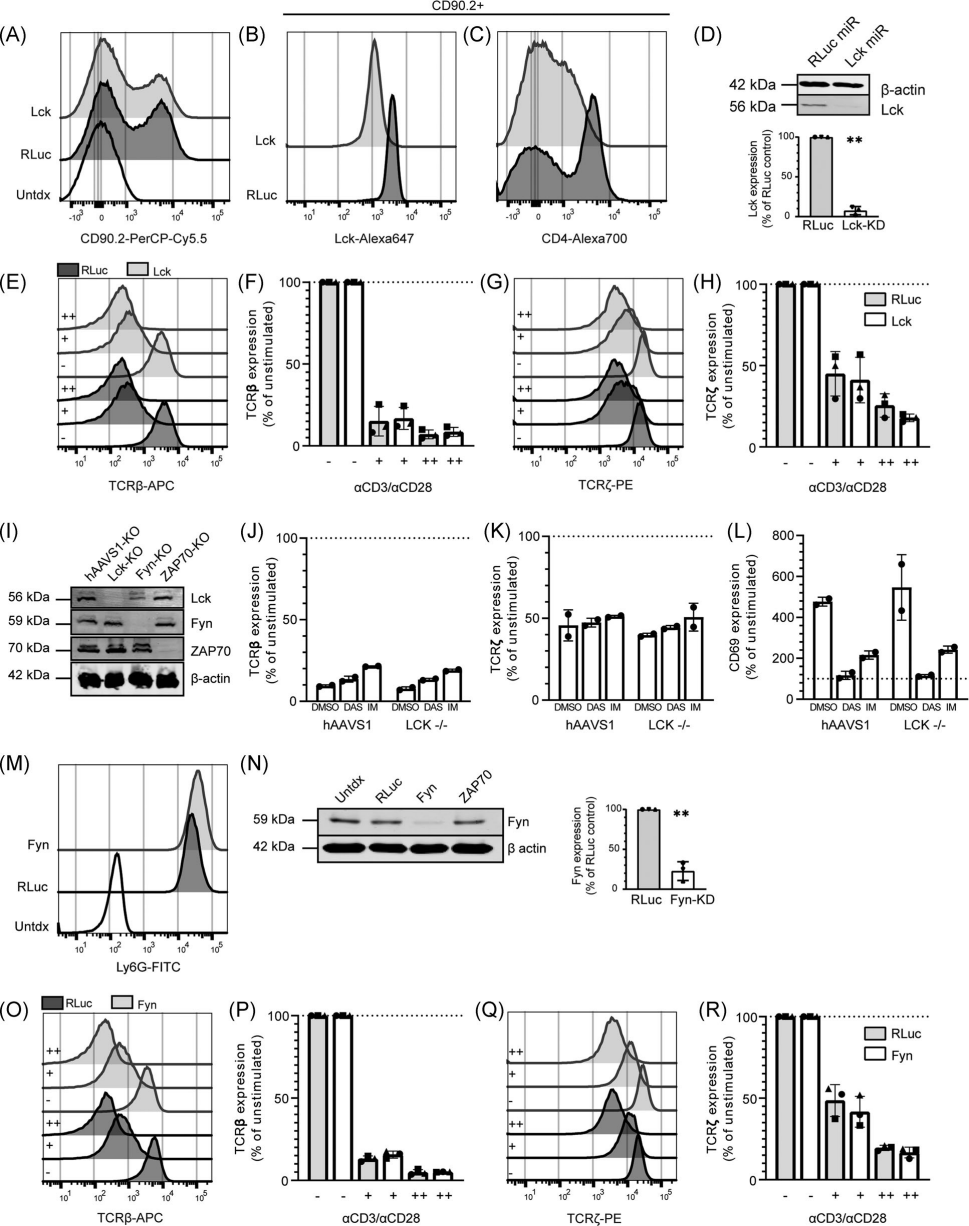
Lck and Fyn are individually redundant for TCR downregulation in human PBLs. (A–D) Transduction efficiency of nontarget microRNA (dark grey) or Lck microRNA vectors (light grey) was measured by CD90.2 (A) expression, and total Lck (B) and surface CD4 (C) expression was measured within the CD90.2^+^ population of human PBLs. (D) Lck knockdown was confirmed and quantified with immunoblot (representative example of *n* = 3 donors). (E–H) Transduced cells were left unstimulated (−), or stimulated with a low (+) or high (++) dose of anti‐CD3/CD28 and surface TCRβ (E, F) and total TCRζ (G, H) was measured. (I) Immunoblots showing the total Lck, Fyn, ZAP70, and β‐actin expression in Jurkat cells (representative example of *n* = 2). (J–L) Surface TCRβ (J), total TCRζ (K) and surface CD69 (L) expression in Jurkat cells transduced with hAAVS1 or LCK CRISPR vectors stimulated with dimethyl sulfoxide, dasatinib or imatinib. Each symbol represents a separate experiment (*n* = 2). Expression in nontriggered T cells is set at 100%, based on the MFI. (M) Transduction efficiency of nontarget microRNA (dark grey) or Fyn microRNA vectors (light grey) was measured by Ly6G. (N) Immunoblots showing the total Fyn and β‐actin expression within the Ly6G^+^ population (representative of *n* = 3 donors). (O–R) Human PBLs transduced with Fyn microRNAs underwent the same stimulation as described for Lck, and surface TCRβ (O, P) and total TCRζ (Q, R) was measured. (F, H, P, R) Each symbol represents an individual donor (*n* = 3) examined in a separate experiment. MFI, mean fluorescence intensity; PBL, peripheral blood lymphocyte; TCR, T‐cell receptor. Significance is calculated with the paired Student's *t* test. **p < .01

To more precisely investigate the contribution of Lck in TCR downregulation, we transduced PBLs with vectors containing the surface marker CD90.2 and microRNAs targeting Lck (Lck‐KD), and assessed the contribution of Lck in TCR downregulation in PBLs. The transduction and knockdown was efficient, as represented by expression of surface CD90.2 (Figure 4A) and total Lck (approximately 70% KD), respectively (Figures 4B and S2A). As expected, Lck knockdown also led to CD4 downregulation, confirming effective depletion of Lck^49^ (Figure 4C). Knockdown was also confirmed with immunoblot (Figure 4D, *p* < .01, approximately 92% KD). Next, the Lck‐KD cells were stimulated with plate‐bound anti‐CD3/CD28 antibodies to examine TCR downregulation. No difference in surface TCRβ downregulation and TCRζ degradation between the nontarget microRNA and the Lck‐KD cells was detected (Figure 4E–H). In contrast, CD69 upregulation was significantly impaired in the Lck‐KD cells (Figure S2B,C; *p* < .01). Similarly, gene knockout of Lck in Jurkat cells by CRISPR/Cas9 did not impair TCR downregulation (Figure S2D–G) even though Lck knockout was complete according to immunoblotting (Figure 4I). The Lck‐KO Jurkat cells had only a 80% lower Lck expression by flow cytometry, which is likely due to background levels of the antibody in flow cytometry (Figure S2D). Lck knockout in Jurkat cells strongly impaired tyrosine phosphorylation, both at baseline and after stimulation with anti‐CD3 (Figure S2H), suggesting successful disruption of Lck. The Lck‐KO cells were stimulated with anti‐CD3/CD28 to investigate the effect on TCR downregulation and CD69 upregulation. Surprisingly, in contrast to the PBLs, this did not affect CD69 upregulation; however, similar to the PBL knockdown TCR downregulation was unaffected (Figure S2E–G). To investigate the effect of PTK inhibitors on TCR downregulation of Jurkat cells, nontarget gRNA or Lck‐KO Jurkat cells were stimulated with anti‐CD3/CD28 in the presence of dasatinib or imatinib. In contrast to PBLs, though these inhibitors block CD69 upregulation, they did not impair TCR downregulation (Figure 4J–L). These results underscore that signal transduction in Jurkat cells differs from PBLs, and therefore it is important to study TCR downregulation in primary cells. Furthermore, these data suggest that the effect of imatinib on Jurkat cells is not exclusively mediated by its action on Lck but possibly by other Src family kinases. These data could also imply that apart from Src family kinases, another family of kinases is involved in TCR downregulation. Together, these data confirm that Lck is required for T cell activation of both primary human T cells and Jurkat cells, but is not essential for full TCR downregulation.

Fyn is another important tyrosine kinase in T cells,^15^ and Fyn and Lck have overlapping functions.^6^, ^15^, ^50^, ^51^ However, the contribution of Fyn to T cell activation and TCR downregulation in primary human T cells is unclear. Therefore, we transduced PBLs with vectors containing the surface marker Ly6G and microRNAs targeting Fyn (Fyn‐KD) (Figure 4M,N and S2I,J). Fyn‐KD did not inhibit TCR downregulation (Figure 4O–R), despite Fyn knockdown being highly efficient (approximately 90% KD, based on immunoblot). Though not significant, a trend of impaired CD69 upregulation was suggested in the Fyn‐KD cells (Figure S2K,L). However, despite efficient knockdown of Fyn we conclude that its role in T cell activation is less pronounced than Lck, which is in accordance with data obtained in mice.^15^, ^29^, ^51^, ^52^ Together, these data suggest that Lck and Fyn are individually dispensable for TCR downregulation, but that especially Lck is essential for full T cell activation.

### 3.7 ZAP70 is dispensable for ligand‐induced TCR downregulation, but not for T cell activation

**Figure 5:**
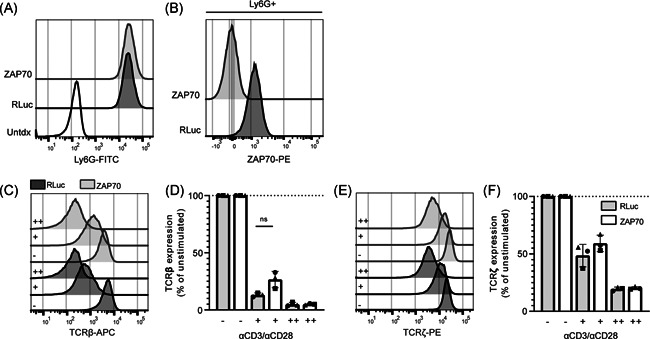
ZAP70 is dispensable for ligand‐induced TCR downregulation in human CD4^+^ PBLs. Human PBLs were transduced with retroviral nontarget microRNA (dark grey), or microRNA vectors targeting ZAP70 (light grey). After transduction, Ly6G (A), and total ZAP70 (B) expression was measured; representative histograms are depicted. Transduced cells were left unstimulated (−), or stimulated with a low (+) or high (++) dose of anti‐CD3/CD28 and surface TCRβ (C, D) and total TCRζ (E, F) expression was measured. (C–E) Representative histograms where dark grey histograms indicate nontarget microRNAs and light grey histograms indicate ZAP70 microRNAs. (D, F) Each symbol represents an individual donor (*n* = 3) examined in a separate experiment, with bars depicting the mean and standard deviations. PBL, peripheral blood lymphocytes; TCR, T‐cell receptor. Significance is calculated with the paired Student's *t* test

Next we investigated the role of ZAP70, as ZAP70 has been suggested to play a nonredundant role in both TCR activation and downregulation.^31^ ZAP70 was knocked down in PBLs with microRNAs. Expression levels of ZAP70 were strongly reduced in the ZAP70‐KD cells (approximately 99% KD) (Figures 5A,B and S3A). ZAP70 knockdown reduced CD69 upregulation (Figure S3B,C) and impaired the production of IL‐2 and IFN‐γ upon stimulation with anti‐CD3/CD28 (Figure S3D). However, analogous to Lck and Fyn, ZAP70 knockdown did not prevent TCR downregulation (Figure 5C–F). Similarly, knockout of ZAP70 in Jurkat cells had no significant effect on TCR downregulation, whereas it impaired CD69 upregulation (Figure S3E–H). Thus, these data suggest that ZAP70 is not essential for TCR downregulation.

### 3.8 Concomitant knockdown of Lck and Fyn modestly impairs ligand‐induced TCR downregulation in T cells

**Figure 6:**
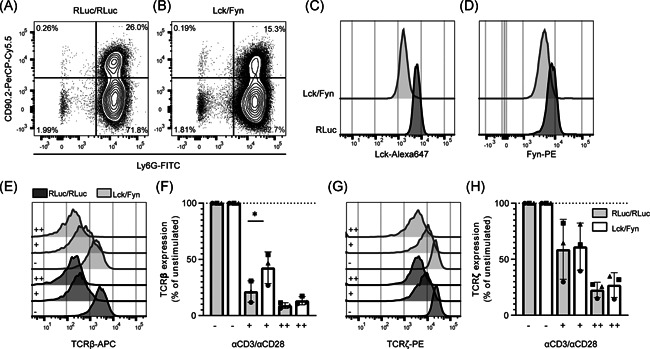
Lck and Fyn double knockdown modestly impairs TCR downregulation in human CD4^+^ PBLs. Representative histograms of surface CD90.2 (A) and Ly6G (B) expression, and total Lck (C) and Fyn (D) expression human PBLs transduced with retroviral nontarget microRNA vectors (dark grey), or microRNA vectors targeting Lck and Fyn (light grey). Representative histograms (E,G) and aggregate data (F,H) of surface TCRβ (E,F) and total TCRζ (G,H) expression in transduced cells left unstimulated (−), or stimulated with a low (+) or high (++) dose of anti‐CD3/CD28. (F,H) Each symbol represents an individual donor (*n* = 3) in a separate experiment, with bars depicting the mean and standard deviations. PBL, peripheral blood lymphocytes; TCR, T‐cell receptor. Significance was calculated with the paired Student's *t* test. *p < .05

On the basis of data obtained in mice, where depleting Lck and Fyn simultaneously strongly impaired T‐cell function,^6^, ^15^, ^51^ we investigated the effect of simultaneously knocking down Lck and Fyn in PBLs (Figure 6A–D and S4A). Lck/Fyn double knockdown (dKD) cells (CD90.2^+^ Ly6G^+^) were stimulated with plate‐bound anti‐CD3/CD28 antibodies, and TCR downregulation as well as cytokine production was assessed. Although Lck/Fyn dKD cells did not show altered TCR downregulation in response to stimulation with a high dose of anti‐CD3/CD28 (Figure 6E,F), significantly impaired TCRβ downregulation was observed in cells stimulated with a lower anti‐CD3/CD28 dose. Notably, this effect was not observed for TCRζ levels (Figure 6G,H). Lck/Fyn dKD also reduced CD69 upregulation (Figure S4B,C) and decreased cytokine production after anti‐CD3/CD28 stimulation (Figure S4D). Thus, despite effective dKD, TCR downregulation was only modestly affected. Together, these data strongly suggest an important role for Lck and Fyn in T cell activation and effector functions. However, the incomplete abrogation of TCR downregulation and the finding that stronger TCR triggering overrides this phenotype imply that other signaling events are involved.

## 4 DISCUSSION

Here, we optimized the methods to genetically modify and stimulate human primary T cells and investigate the mechanisms of TCR downregulation. With PTK inhibitors, we strongly inhibited TCR downregulation, and thereby suggest that Src family kinases as a group are involved in TCR downregulation in human PBLs. However, using genetic modification, we found that individual silencing of Lck, Fyn or ZAP70 did not inhibit TCR downregulation. Lck and Fyn dKD partially inhibited TCR downregulation, but did not fully block this process. These data suggest that Lck and Fyn have important yet partially redundant roles in TCR downregulation.

Multiple studies have investigated the role of kinase function in TCR downregulation in murine lymphocytes or cell lines, but the subject has remained unresolved due to conflicting data.^23^, ^53^, ^54^, ^55^ These previous studies were mostly performed with PTK inhibitors such as genistein, herbimycin, or tyrphostin. In comparison, the newer generation or optimized PTK inhibitors PP2, dasatinib and imatinib used here, are more efficient and specific. Of these compounds, PP2 and dasatinib are wide‐range PTK inhibitors that fully blocked T cell activation and strongly impaired TCR downregulation in our model. In contrast, imatinib is postulated to be more specific in its inhibition, with Lck as the sole predicted target in human T cells.^44^, ^45^, ^56^ Interestingly, we observed significant inhibition of T cell activation and TCRζ degradation with either PP2, dasatinib or imatinib, but the inhibition of T cell activation and TCRβ downregulation was less pronounced with the latter. On the basis of literature we initially hypothesized that imatinib acts specifically on Lck, whereas PP2 and dasatinib have a stronger phenotypic effect, because they inhibit other nonredundant kinases besides Lck. However, our findings do not directly support that Lck is the main target of imatinib and dasatinib in our systems. First, dasatinib and imatinib block TCR downregulation to a similar extent in wild‐type Jurkat cells compared to Lck‐KO cells. Second, we did not observe similar phenotypes in Lck‐KD/Lck‐KO compared to imatinib treated cells in either the Jurkat or PBL experiments. The different phenotypes resulting from imatinib and dasatinib treatment could alternatively be explained by a less‐potent inhibition of its target by imatinib compared to dasatinib. Concurrently, there could be an even wider network of kinases partially involved in TCR downregulation, which are inhibited by dasatinib and PP2, but not by imatinib. Future research will have to address the validity of these hypotheses.

The importance of Lck in T‐cell development, T‐cell activation, and TCR downregulation has been extensively studied. It has been established that TCR downregulation is an essential part of thymocyte development.^57^, ^58^ Cumulative evidence from data in murine thymocytes, Jurkat cells, and artificial overexpression studies suggested that Lck is an essential kinase in TCR activation and downregulation.^16^, ^30^, ^59^ Contrary to these results, we find no evidence that Lck is individually required for TCR downregulation in either CRISPR/Cas9‐modified Jurkat cells or in modified human PBLs. Therefore, we also investigated the kinase Fyn, whose function is similar to Lck. However, also in Fyn‐KD cells, we did not observe impaired TCR downregulation, which concurs with the hypothesis of individual redundancy. A contribution of Lck and Fyn together would be in line with data obtained from mouse models investigating T cell development.^29^, ^52^ Furthermore, Lck and Fyn also function in concert during T cell activation in mice.^15^, ^51^, ^60^ In concordance with these mouse studies, we found a small but significant inhibition of TCR downregulation and CD69 upregulation in human PBLs with Lck and Fyn knockdown. However, our results simultaneously clearly show that TCR downregulation still occurred to a great extent despite the efficient knockdown of both Lck and Fyn, implying that these kinases are relatively more redundant for ligand‐induced TCR downregulation in PBLs than for tonic TCR downregulation in thymocytes. Therefore, we propose that other protein kinases may be involved in TCR downregulation, which could include kinases from other families.

We further hypothesized that inhibition of ZAP70 would interfere with TCR downregulation, as Dumont et al.^31^ previously observed a modest phenotype in patients with congenital ZAP70 deficiencies. ZAP70 is generally thought to be essential to relay activating signals from TCR triggering mediated by Lck and Fyn^61^, ^62^ and we expected strong inhibition of T cell activation and TCR downregulation when inhibiting this nonredundant downstream‐signaling molecule. Indeed, we observed abrogation of T‐cell effector functions in the form of cytokine production and CD69 upregulation in response to ZAP70 knockdown. Despite the importance of ZAP70 in T cell activation and cytokine production, we found that its role is individually dispensable for TCR downregulation. The discrepancy between our observations and Dumont et al.^31^ could be explained by the method and moment of ZAP70 depletion. TCR downregulation in T cells from donors with a congenital ZAP70 deficiency may be subject to compensatory mechanisms, whereas depletion of ZAP70 in circulating PBLs circumvents this issue.

It should be noted that all that our results are obtained with anti‐CD3/CD28 antibody‐mediated TCR triggering, and more physiologic stimulation with peptide:MHC triggering and native costimulation could present alternative results. Our data suggests that TCR downregulation occurs relatively early after stimulation, whereas CD69 upregulation usually takes longer. Therefore, we stimulated the T cells with anti‐CD3/CD28 for 16 h, and measured TCR downregulation and CD69 upregulation simultaneously. We can hereby exclude the duration of stimulation as a factor that influences the discrepancy between TCR downregulation and CD69 upregulation, suggesting that the differences observed are due to effective knockdown. Finally, our results are obtained in T cells that are activated at least 1 week before the functional experiments. At the moment of functional assays (7–10 days postactivation), T cells had reverted to their original size and CD69 expression levels, but we cannot exclude that the previous activation of these T cells affects their signaling upon recurrent stimulation. It is possible that a partial inhibition by inhibitors or microRNA knockdown of Lck, Fyn or ZAP70 inhibits T cell activation but allows TCR downregulation, because TCR downregulation would require a lower phosphorylation or signaling threshold. Although we cannot fully exclude this hypothesis, our data showing that low‐dose anti‐CD3/CD28 stimulation induces less TCR downregulation, but similar CD69 upregulation, argue against this. Together, our data also underscore the profound differences between Jurkat cells and human PBLs, for instance when interpreting the effect of PTK inhibitors on TCR downregulation, or CD69 upregulation in the different models. This illustrates the importance of reinvigorating the mechanistic studies of TCR downregulation in primary cells, now that the genetic toolbox has expanded.

In summary, this study has made clear that TCR downregulation is a process with marked differences between formerly used mouse and cell‐line models, and human PBLs. Although tremendous progress has been made to understand the molecular pathways of T‐cell signaling in recent decades, research on the mechanisms of TCR downregulation has lagged behind.^1^, ^63^ The importance of a better understanding of TCR downregulation is apparent, as it has direct clinical implications for the understanding of both autoimmune disorders as well as the improvement of immune therapies by increasing the reactivity of T cells. In this light it is important to note that we find clear evidence of lower TCR levels in antigen‐experienced PBLs isolated from healthy donors. This observation supports recent findings obtained in mice, which showed that TCR downregulation in vivo can persist for long periods of time and is likely a mechanism to fine‐tune T‐cell responses to antigens with varying affinities.^22^ Together, this study highlights the importance and complexity of studying TCR downregulation in human primary T cells. Though we show that Src family kinases drive this process, we emphasize that more research is needed to understand the individual molecules and pathways involved. Our study provides useful tools to answer these questions.

## CONFLICT OF INTERESTS

The authors declare that there are no conflict of interests.

## AUTHOR CONTRIBUTIONS

*Design and interpretation of experiments*: Lieve E. H. van der Donk, Louis S. Ates, and Jeroen W. J. van Heijst. *Performance of experiments*: Lieve E. H. van der Donk, Jet van der Spek, and Laura M. Tukker. *Manuscript writing*: Lieve E. H. van der Donk, Louis S. Ates, Teunis B. H. Geitenbeek, and Jeroen W. J. van Heijst.

## Supporting information

Supporting information.

## Data Availability

The data that support the findings of this study are available upon reasonable request.

## References

[1] Alcover A , Alarcon B , Di Bartolo V. Cell biology of T‐cell receptor expression and regulation. Annu Rev Immunol. 2018;36:103‐125. 29261409 10.1146/annurev-immunol-042617-053429

[2] Wucherpfennig KW , Gagnon E , Call MJ , Huseby ES , Call ME . Structural biology of the T‐cell receptor: insights into receptor assembly, ligand recognition, and initiation of signaling. Cold Spring Harb Perspect Biol. 2010;2(4):a005140. 20452950 10.1101/cshperspect.a005140 PMC2845206

[3] Latour S , Veillette A. Proximal protein tyrosine kinases in immunoreceptor signaling. Curr Opin Immunol. 2001;13(3):299‐306. 11406361 10.1016/s0952-7915(00)00219-3

[4] Chakraborty AK , Weiss A. Insights into the initiation of TCR signaling. Nature Immunol. 2014;15(9):798‐807. 25137454 10.1038/ni.2940 PMC5226627

[5] Palacios EH , Weiss A. Function of the Src‐family kinases, Lck and Fyn, in T‐cell development and activation. Oncogene. 2004;23(48):7990‐8000. 15489916 10.1038/sj.onc.1208074

[6] Denny MF , Patai B , Straus DB . Differential T‐cell antigen receptor signaling mediated by the Src family kinases Lck and Fyn. Mol Cell Biol. 2000;20(4):1426‐1435. 10648627 10.1128/mcb.20.4.1426-1435.2000 PMC85301

[7] James JR , Vale RD . Biophysical mechanism of T‐cell receptor triggering in a reconstituted system. Nature. 2012;487(7405):64‐69. 22763440 10.1038/nature11220 PMC3393772

[8] Su X , Ditlev JA , Hui E , et al. Phase separation of signaling molecules promotes T‐cell receptor signal transduction. Science. 2016;352(6285):595‐599. 27056844 10.1126/science.aad9964 PMC4892427

[9] Veillette A , Bookman MA , Horak EM , Bolen JB . The CD4 and CD8 T cell surface antigens are associated with the internal membrane tyrosine‐protein kinase p56lck. Cell. 1988;55(2):301‐308. 3262426 10.1016/0092-8674(88)90053-0

[10] Artyomov MN , Lis M , Devadas S , Davis MM , Chakraborty AK . CD4 and CD8 binding to MHC molecules primarily acts to enhance Lck delivery. Proc Natl Acad Sci USA. 2010;107(39):16916‐16921. 20837541 10.1073/pnas.1010568107 PMC2947881

[11] van Oers NS , Killeen N , Weiss A. Lck regulates the tyrosine phosphorylation of the T‐cell receptor subunits and ZAP‐70 in murine thymocytes. J Exp Med. 1996;183(3):1053‐1062. 8642247 10.1084/jem.183.3.1053 PMC2192313

[12] Thill PA , Weiss A , Chakraborty AK . Phosphorylation of a tyrosine residue on Zap70 by Lck and its subsequent binding via an SH2 domain may be a key gatekeeper of T‐cell receptor signaling in vivo. Mol Cell Biol. 2016;36(18):2396‐2402. 27354065 10.1128/MCB.00165-16 PMC5007795

[13] Zhang W , Sloan‐Lancaster J , Kitchen J , Trible RP , Samelson LE . LAT: the ZAP‐70 tyrosine kinase substrate that links T‐cell receptor to cellular activation. Cell. 1998;92(1):83‐92. 9489702 10.1016/s0092-8674(00)80901-0

[14] Wardenburg JB , Fu C , Jackman JK , et al. Phosphorylation of SLP‐76 by the ZAP‐70 protein‐tyrosine kinase is required for T‐cell receptor function. J Biol Chem. 1996;271(33):19641‐19644. 8702662 10.1074/jbc.271.33.19641

[15] Lovatt M , Filby A , Parravicini V , Werlen G , Palmer E , Zamoyska R. Lck regulates the threshold of activation in primary T cells, while both Lck and Fyn contribute to the magnitude of the extracellular signal‐related kinase response. Mol Cell Biol. 2006;26(22):8655‐8665. 16966372 10.1128/MCB.00168-06 PMC1636771

[16] D'Oro U , Vacchio MS , Weissman AM , Ashwell JD . Activation of the Lck tyrosine kinase targets cell surface T cell antigen receptors for lysosomal degradation. Immunity. 1997;7(5):619‐628. 9390686 10.1016/s1074-7613(00)80383-0

[17] Liu H , Rhodes M , Wiest DL , Vignali DA . On the dynamics of TCR:CD3 complex cell surface expression and downmodulation. Immunity. 2000;13(5):665‐675. 11114379 10.1016/s1074-7613(00)00066-2

[18] Cenciarelli C , Hou D , Hsu K , et al. Activation‐induced ubiquitination of the T cell antigen receptor. Science. 1992;257(5071):795‐797. 1323144 10.1126/science.1323144

[19] Rubin B , Llobera R , Gouaillard C , Alcover A , Arnaud J. Dissection of the role of CD3gamma chains in profound but reversible T‐cell receptor down‐regulation. Scand J Immunol. 2000;52(2):173‐183. 10931385 10.1046/j.1365-3083.2000.00767.x

[20] Itoh Y , Hemmer B , Martin R , Germain RN . Serial TCR engagement and down‐modulation by peptide:MHC molecule ligands: relationship to the quality of individual TCR signaling events. J Immunol. 1999;162(4):2073‐2080. 9973480

[21] Valitutti S , Muller S , Cella M , Padovan E , Lanzavecchia A. Serial triggering of many T‐cell receptors by a few peptide‐MHC complexes. Nature. 1995;375(6527):148‐151. 7753171 10.1038/375148a0

[22] Gallegos AM , Xiong H , Leiner IM , et al. Control of T cell antigen reactivity via programmed TCR downregulation. Nature Immunol. 2016;17(4):379‐386. 26901151 10.1038/ni.3386 PMC4803589

[23] Cai Z , Kishimoto H , Brunmark A , Jackson MR , Peterson PA , Sprent J. Requirements for peptide‐induced T‐cell receptor downregulation on naive CD8+ T cells. J Exp Med. 1997;185(4):641‐651. 9034143 10.1084/jem.185.4.641 PMC2196147

[24] Schönrich G , Kalinke U , Momburg F , et al. Down‐regulation of T‐cell receptors on self‐reactive T cells as a novel mechanism for extrathymic tolerance induction. Cell. 1991;65(2):293‐304. 1849799 10.1016/0092-8674(91)90163-s

[25] Valitutti S , Lanzavecchia A. Serial triggering of TCRs: a basis for the sensitivity and specificity of antigen recognition. Immunol Today. 1997;18(6):299‐304. 9190117

[26] Viola A , Lanzavecchia A. T cell activation determined by T‐cell receptor number and tunable thresholds. Science. 1996;273(5271):104‐106. 8658175 10.1126/science.273.5271.104

[27] Myers MD , Dragone LL , Weiss A. Src‐like adaptor protein down‐regulates T‐cell receptor (TCR)‐CD3 expression by targeting TCRzeta for degradation. J Cell Biol. 2005;170(2):285‐294. 16027224 10.1083/jcb.200501164 PMC2171412

[28] Straus DB , Weiss A. Genetic evidence for the involvement of the lck tyrosine kinase in signal transduction through the T cell antigen receptor. Cell. 1992;70(4):585‐593. 1505025 10.1016/0092-8674(92)90428-f

[29] van Oers NS , Lowin‐Kropf B , Finlay D , Connolly K , Weiss A. alpha beta T cell development is abolished in mice lacking both Lck and Fyn protein tyrosine kinases. Immunity. 1996;5(5):429‐436. 8934570 10.1016/s1074-7613(00)80499-9

[30] Lauritsen JP , Christensen MD , Dietrich J , Kastrup J , Odum N , Geisler C. Two distinct pathways exist for down‐regulation of the TCR. J Immunol. 1998;161(1):260‐267. 9647232

[31] Dumont C , Blanchard N , Di Bartolo V , et al. TCR/CD3 down‐modulation and zeta degradation are regulated by ZAP‐70. J Immunol. 2002;169(4):1705‐1712. 12165490 10.4049/jimmunol.169.4.1705

[32] von Essen M , Bonefeld CM , Siersma V , et al. Constitutive and ligand‐induced TCR degradation. The Journal of Immunology. 2004;173(1):384‐393. 15210797 10.4049/jimmunol.173.1.384

[33] Bartelt RR , Cruz‐Orcutt N , Collins M , Houtman JC . Comparison of T‐cell receptor‐induced proximal signaling and downstream functions in immortalized and primary T cells. PLOS One. 2009;4(5):e5430. 19412549 10.1371/journal.pone.0005430 PMC2673025

[34] Kitamura T , Koshino Y , Shibata F , et al. Retrovirus‐mediated gene transfer and expression cloning: powerful tools in functional genomics. Exp Hematol. 2003;31(11):1007‐1014. 14585362

[35] Hart T , Tong AHY , Chan K , et al. Evaluation and design of genome‐wide CRISPR/SpCas9 knockout screens. G3 (Bethesda). 2017;7(8):2719‐2727. 28655737 10.1534/g3.117.041277 PMC5555476

[36] Naviaux RK , Costanzi E , Haas M , Verma IM . The pCL vector system: rapid production of helper‐free, high‐titer, recombinant retroviruses. J Virol. 1996;70(8):5701‐5705. 8764092 10.1128/jvi.70.8.5701-5705.1996 PMC190538

[37] Chang K , Marran K , Valentine A , Hannon GJ . Packaging shRNA retroviruses. Cold Spring Harb Protoc. 2013;2013(8):734‐737. 23906912 10.1101/pdb.prot076448

[38] Salmon P , Trono D. Production and titration of lentiviral vectors. Curr Protoc Hum Genet. 2007;54(1). 10.1002/0471142905.hg1210s54 18428406

[39] Luton F , Buferne M , Legendre V , Chauvet E , Boyer C , Schmitt‐Verhulst AM . Role of CD3gamma and CD3delta cytoplasmic domains in cytolytic T lymphocyte functions and TCR/CD3 down‐modulation. J Immunol. 1997;158(9):4162‐4170. 9126976

[40] Kishimoto H , Kubo RT , Yorifuji H , Nakayama T , Asano Y , Tada T. Physical dissociation of the TCR‐CD3 complex accompanies receptor ligation. J Exp Med. 1995;182(6):1997‐2006. 7500045 10.1084/jem.182.6.1997 PMC2192252

[41] Valitutti S , Muller S , Salio M , Lanzavecchia A. Degradation of T‐cell receptor (TCR)‐CD3‐zeta complexes after antigenic stimulation. J Exp Med. 1997;185(10):1859‐1864. 9151711 10.1084/jem.185.10.1859 PMC2196323

[42] Salmond RJ , Filby A , Qureshi I , Caserta S , Zamoyska R. T‐cell receptor proximal signaling via the Src‐family kinases, Lck and Fyn, influences T‐cell activation, differentiation, and tolerance. Immunol Rev. 2009;228(1):9‐22. 19290918 10.1111/j.1600-065X.2008.00745.x

[43] Hanke JH , Gardner JP , Dow RL , et al. Discovery of a novel, potent, and Src family‐selective tyrosine kinase inhibitor. Study of Lck‐ and FynT‐dependent T cell activation. J Biol Chem. 1996;271(2):695‐701. 8557675 10.1074/jbc.271.2.695

[44] Lee KC , Ouwehand I , Giannini AL , Thomas NS , Dibb NJ , Bijlmakers MJ . Lck is a key target of imatinib and dasatinib in T‐cell activation. Leukemia. 2010;24(4):896‐900. 20147973 10.1038/leu.2010.11

[45] Cwynarski K , Laylor R , Macchiarulo E , et al. Imatinib inhibits the activation and proliferation of normal T lymphocytes in vitro. Leukemia. 2004;18(8):1332‐1339. 15190258 10.1038/sj.leu.2403401

[46] Wang W , Ye C , Liu J , Zhang D , Kimata JT , Zhou P. CCR5 gene disruption via lentiviral vectors expressing Cas9 and single guided RNA renders cells resistant to HIV‐1 infection. PLOS One. 2014;9(12):e115987. 25541967 10.1371/journal.pone.0115987 PMC4277423

[47] Seki A , Rutz S. Optimized RNP transfection for highly efficient CRISPR/Cas9‐mediated gene knockout in primary T cells. J Exp Med. 2018;215(3):985‐997. 29436394 10.1084/jem.20171626 PMC5839763

[48] Dietrich J , Geisler C. T‐cell receptor zeta allows stable expression of receptors containing the CD3gamma leucine‐based receptor‐sorting motif. J Biol Chem. 1998;273(41):26281‐26284. 9756853 10.1074/jbc.273.41.26281

[49] Pelchen‐Matthews A , Boulet I , Littman DR , Fagard R , Marsh M. The protein tyrosine kinase p56lck inhibits CD4 endocytosis by preventing entry of CD4 into coated pits. J Cell Biol. 1992;117(2):279‐290. 1373141 10.1083/jcb.117.2.279 PMC2289416

[50] Chan AC , Dalton M , Johnson R , et al. Activation of ZAP‐70 kinase activity by phosphorylation of tyrosine 493 is required for lymphocyte antigen receptor function. EMBO J. 1995;14(11):2499‐2508. 7781602 10.1002/j.1460-2075.1995.tb07247.x PMC398363

[51] Tsun A , Qureshi I , Stinchcombe JC , et al. Centrosome docking at the immunological synapse is controlled by Lck signaling. J Cell Biol. 2011;192(4):663‐674. 21339332 10.1083/jcb.201008140 PMC3044125

[52] Groves T , Smiley P , Cooke MP , Forbush K , Perlmutter RM , Guidos CJ . Fyn can partially substitute for Lck in T lymphocyte development. Immunity. 1996;5(5):417‐428. 8934569 10.1016/s1074-7613(00)80498-7

[53] Luton F , Buferne M , Davoust J , Schmitt‐Verhulst AM , Boyer C. Evidence for protein tyrosine kinase involvement in ligand‐induced TCR/CD3 internalization and surface redistribution. J Immunol. 1994;153(1):63‐72. 8207256

[54] Salio M , Valitutti S , Lanzavecchia A. Agonist‐induced T‐cell receptor down‐regulation: molecular requirements and dissociation from T cell activation. Eur J Immunol. 1997;27(7):1769‐1773. 9247590 10.1002/eji.1830270726

[55] Martin S , Bevan MJ . Transient alteration of T cell fine specificity by a strong primary stimulus correlates with T‐cell receptor down‐regulation. Eur J Immunol. 1998;28(10):2991‐3002. 9808168 10.1002/(SICI)1521-4141(199810)28:10<2991::AID-IMMU2991>3.0.CO;2-B PMC2782384

[56] Fabian MA , Biggs WH 3rd , Treiber DK , et al. A small molecule‐kinase interaction map for clinical kinase inhibitors. Nat Biotechnol. 2005;23(3):329‐336. 15711537 10.1038/nbt1068

[57] Wang H , Holst J , Woo SR , et al. Tonic ubiquitylation controls T‐cell receptor:CD3 complex expression during T‐cell development. EMBO J. 2010;29(7):1285‐1298. 20150895 10.1038/emboj.2010.10 PMC2857457

[58] Bluestone JA , Pardoll D , Sharrow SO , Fowlkes BJ . Characterization of murine thymocytes with CD3‐associated T‐cell receptor structures. Nature. 1987;326(6108):82‐84. 3102972 10.1038/326082a0

[59] Molina TJ , Kishihara K , Siderovskid DP , et al. Profound block in thymocyte development in mice lacking p56lck. Nature. 1992;357(6374):161‐164. 1579166 10.1038/357161a0

[60] Seddon B , Legname G , Tomlinson P , Zamoyska R. Long‐term survival but impaired homeostatic proliferation of Naive T cells in the absence of p56lck. Science. 2000;290(5489):127‐131. 11021796 10.1126/science.290.5489.127

[61] Chan AC , Iwashima M , Turck CW , Weiss A. ZAP‐70: a 70 kd protein‐tyrosine kinase that associates with the TCR zeta chain. Cell. 1992;71(4):649‐662. 1423621 10.1016/0092-8674(92)90598-7

[62] Williams BL , Schreiber KL , Zhang W , et al. Genetic evidence for differential coupling of Syk family kinases to the T‐cell receptor: reconstitution studies in a ZAP‐70‐deficient Jurkat T‐cell line. Mol Cell Biol. 1998;18(3):1388‐1399. 9488454 10.1128/mcb.18.3.1388 PMC108852

[63] Baniyash M. TCR zeta‐chain downregulation: curtailing an excessive inflammatory immune response. Nat Rev Immunol. 2004;4(9):675‐687. 15343367 10.1038/nri1434

