# Supplementary material for: Separate signaling events control TCR downregulation and T cell activation in primary human T cells
Source: Immun Inflamm Dis. 2020 Dec 22;9(1):223–38. doi: 10.1002/iid3.383 (PMC7860602; doi:10.1002/iid3.383)
Supplement: Supplementary file 1 — Supporting information. [file IID3-9-223-s001.pdf]

Supplemental information

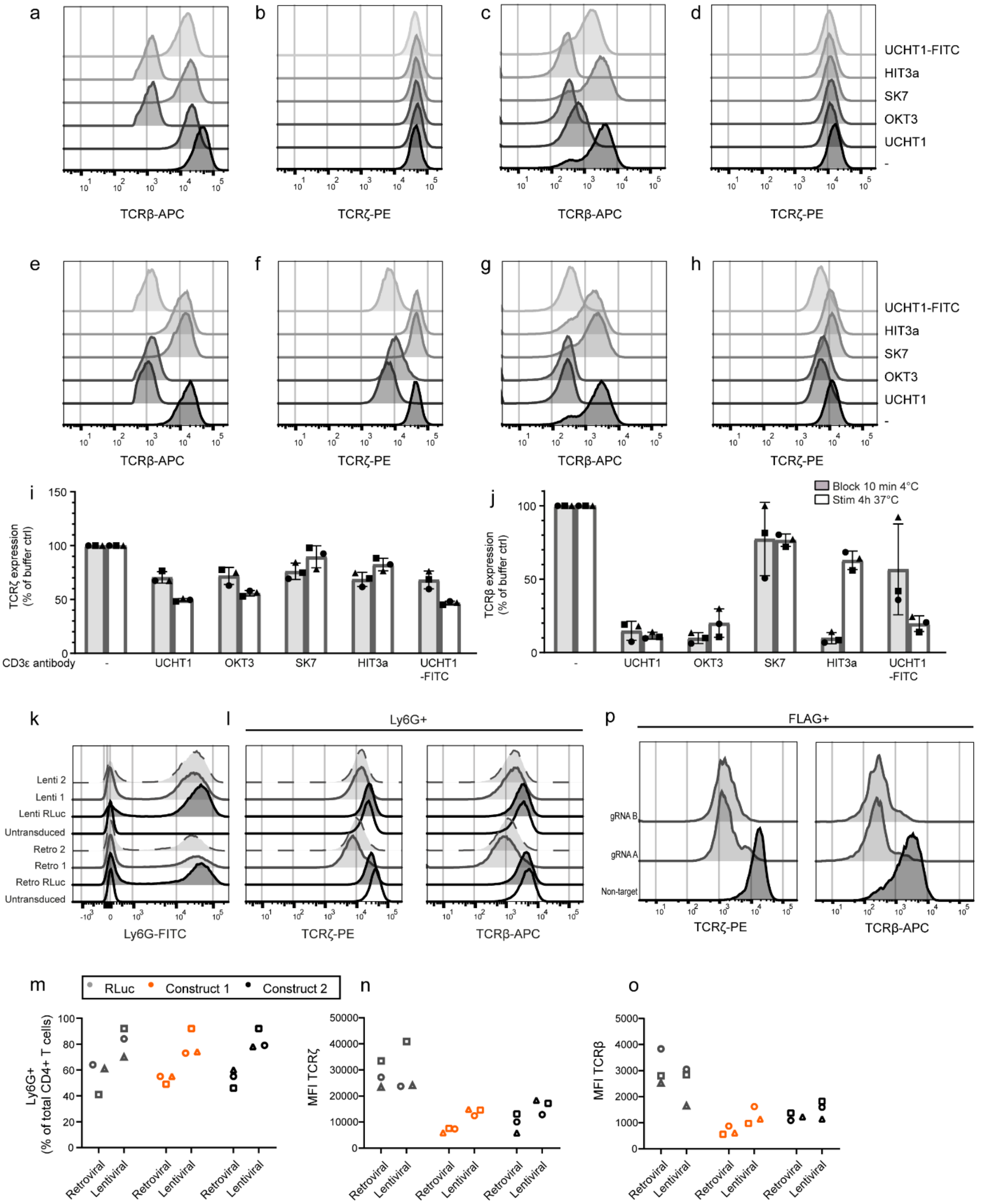

Supplemental Figure 1. Investigation of steric hindrance and stimulation, and genetic modification of human CD4+ PBLs and Jurkat cells

(A-D) Representative histograms showing steric hindrance for human PBLs (A, B) and Jurkat cells (C, D). Surface TCRβ (A) and total TCRζ (B) expression in human PBLs, and surface TCRβ (C) and total TCRζ

(D) expression in Jurkat cells were measured after incubation with the indicated anti-CD3 antibodies at 4°C. (E-H) Representative histograms showing the effect of anti-CD3/CD28 stimulation on human PBLs (E, F) and Jurkat cells (G, H). Surface TCRβ (E) and total TCRζ (F) expression in human PBLs, and surface TCRβ (G) and total TCRζ (H) expression in Jurkat cells after stimulation at 37°C.

(I-J) Aggregate data of total TCRζ (I) and surface TCRβ (J) expression of Jurkat cells. For Jurkat cells, each symbol represents an independent experiment (n=3), and the median and 95% CI are shown. Expression in non-triggered T cells is set at 100%, based on the MFI.

(K-P) Optimization of genetic modification of hPBL and Jurkat cells. (K-L) Representative histograms showing the surface Ly6G (K), total TCRζ, and surface TCRβ (L) expression of human $CD4^+$ PBLs after transduction with retroviral/lentiviral microRNA vectors targeting TCRζ in n=3 independent experiments with a different donor.

(M-O) Comparison between lentiviral and retroviral microRNA knockdown efficiency of TCRζ in human $CD4^+$ PBLs. Percentage of transduced cells ($Ly6G^+$; M), total TCRζ (N) and surface TCRβ (O) levels were measured. (P) For the transduction of Jurkat cells with lentiviral CRISPR/Cas9 vectors targeting TCRζ, total TCRζ and surface TCRβ expression in FLAG-positive Jurkat cells was measured. (M-O) Each symbol represents an individual donor, examined in a separate experiment. (P) Representative plots of n=2 independent experiments.

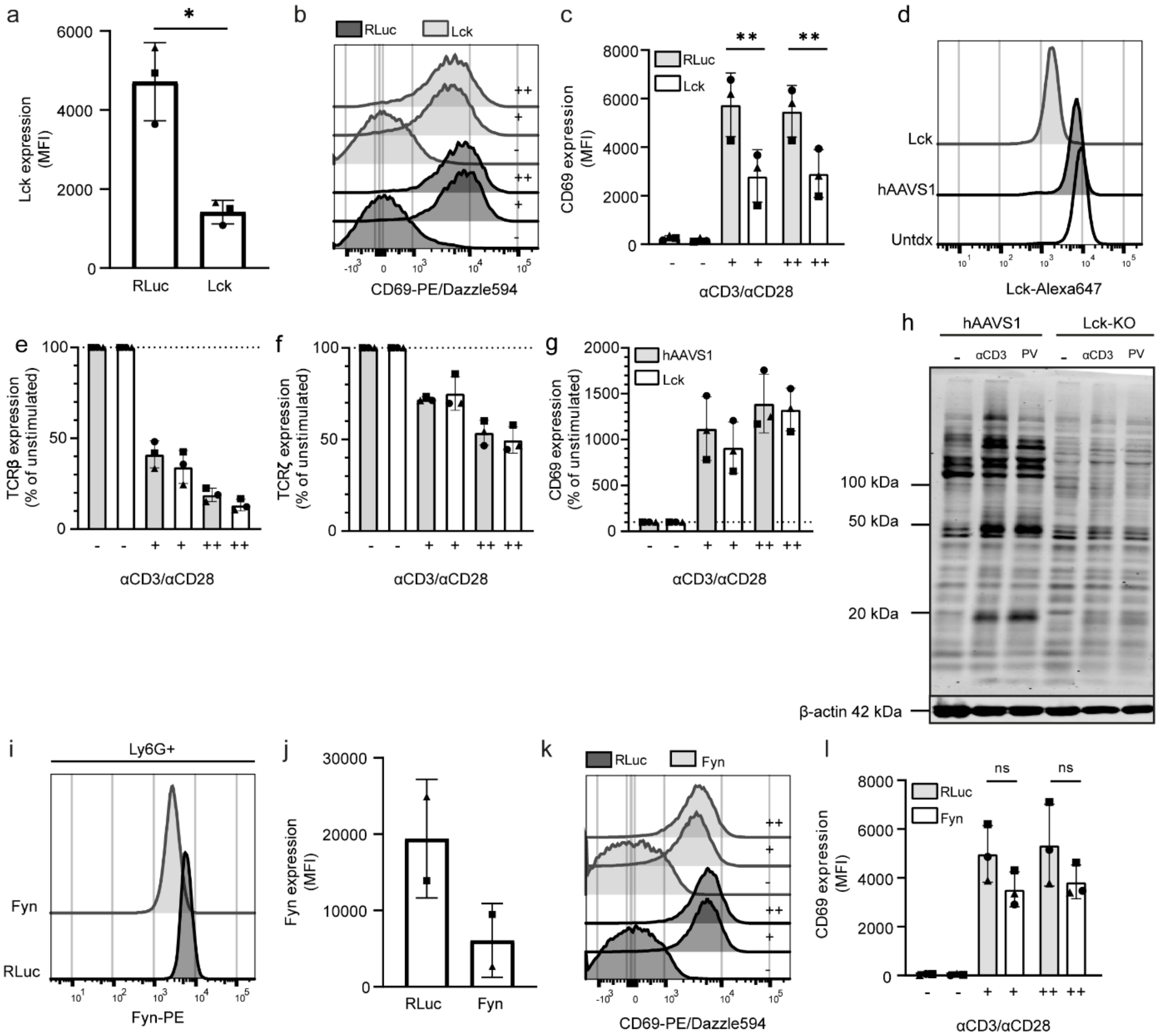

Supplemental Figure 2. Lck and Fyn are individually redundant for TCR downregulation in human PBLs and Jurkat cells

(A) Aggregate data of total Lck expression of human PBLs after transduction with microRNA vectors targeting Lck, for n=3 healthy donors examined in a separate experiment. (B, C) (B) Representative histogram and (C) aggregate data showing the effect of Lck knockdown on CD69 expression in different donors (n=3) and different experiments that were left unstimulated (-), or stimulated with low (+) or high (++) dose anti-CD3/CD28. Significance is calculated with the paired student's *t*-test, *p<0.05; **p<0.01.

(D) Total Lck expression of Jurkat cells after transduction with CRISPR/Cas9 vectors targeting Lck.

(E-G) Surface TCRβ (E), total TCRζ (F) and surface CD69 (G) expression of Jurkat cells with Lck knockdown left unstimulated (-), or upon TCR triggering by low (+) or high (++) dose anti-CD3/CD28 for n=3 separate experiments.

(H) Immunoblot showing the total tyrosine phosphorylation of cells that were transduced with non-target or Lck CRISPR/Cas9 vectors, and that were unstimulated (-), anti-CD3 stimulated, or anti-CD3 and pervanadate (PV) stimulated. β-actin was used as loading control. Immunoblot is representative of a single biological replicate.

(I) Representative histogram showing the total Fyn expression of human PBLs after transduction with non-target microRNAs or microRNA vectors targeting Fyn. (J) Aggregate data of total Fyn expression of human PBLs after transduction with microRNA vectors targeting Fyn, for n=2 healthy donors examined in a separate experiment.

(K, L) Representative histogram (K) and aggregate data (L) showing the effect of Fyn knockdown on CD69 expression in different donors (n=3) that were left unstimulated (-), or stimulated with low (+) or high (++) dose anti-CD3/CD28. Significance is calculated with the paired student's *t*-test, *$p<0.05$; **$p<0.01$.

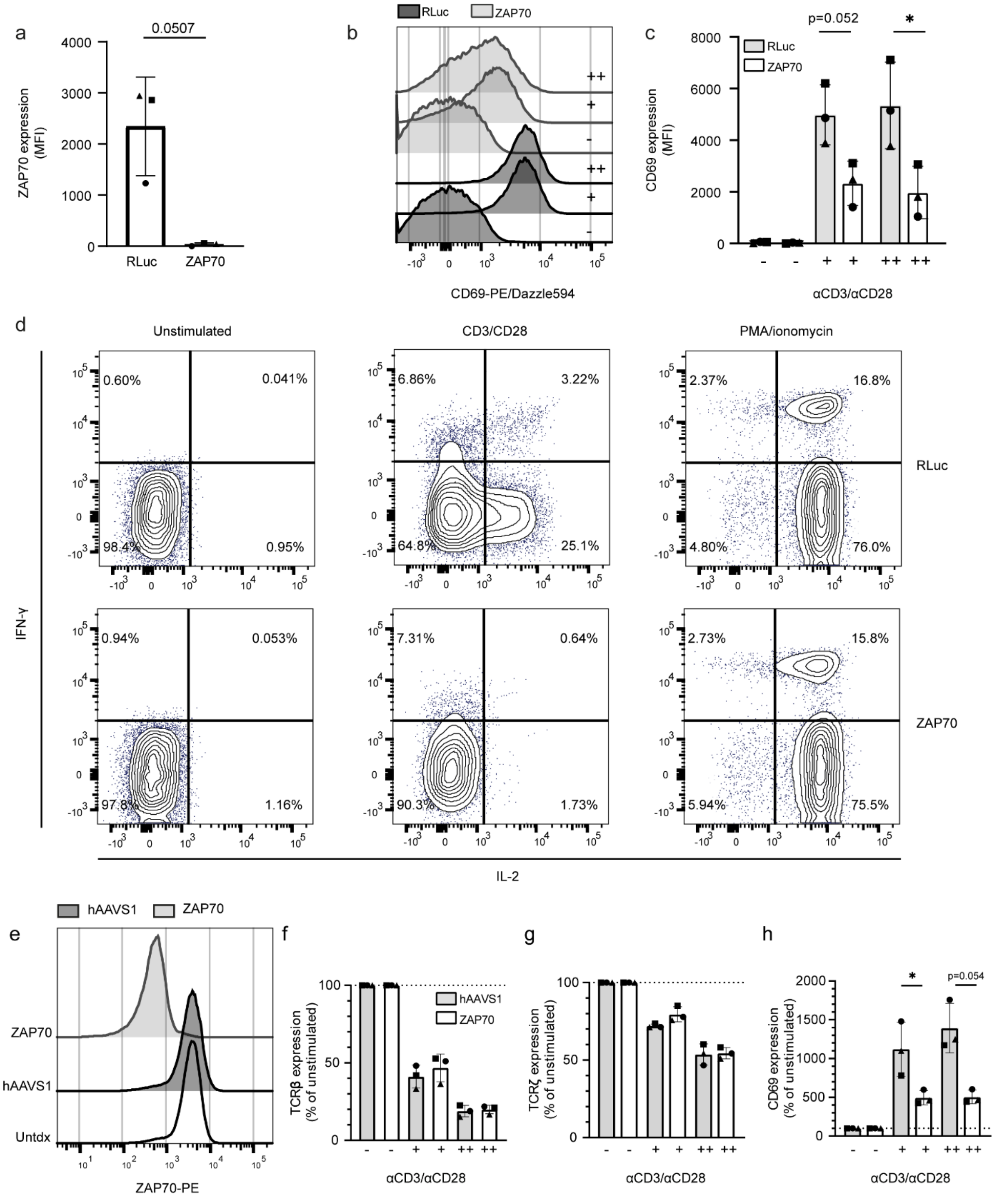

Supplemental Figure 3. ZAP70 is required for T cell activation and effector functions in human CD4$^+$ PBLs

(A) Aggregate data of total ZAP70 expression of human PBLs after transduction with microRNA vectors targeting ZAP70. (B, C) Representative histogram (B) and aggregate data (C) of surface CD69 expression in human PBLs with ZAP70 knockdown (dark grey) or non-target microRNA (light grey)

without stimulation (-), or upon TCR triggering by low (+) or high (++) dose anti-CD3/CD28. (A, C) Each symbol represents an individual donor (n=3) examined in a separate experiment, with bars depicting the mean and standard deviation. Significance was calculated with paired student's *t*-test. *p<0.05. (D) Effect of ZAP70 knockdown on cytokine production by PBLs. Representative plots of 1 donor. (E) Total ZAP70 expression of Jurkat cells after transduction with CRISPR/Cas9 vectors targeting ZAP70 (light grey) or a non-target guide-RNA (dark grey). (F-H) Surface TCRβ (F), total TCRζ (G) and surface CD69 (H) expression of PBLs with ZAP70 knockout left unstimulated (-), or upon TCR triggering by low (+) or high (++) dose anti-CD3/CD28 for n=3 separate experiments.

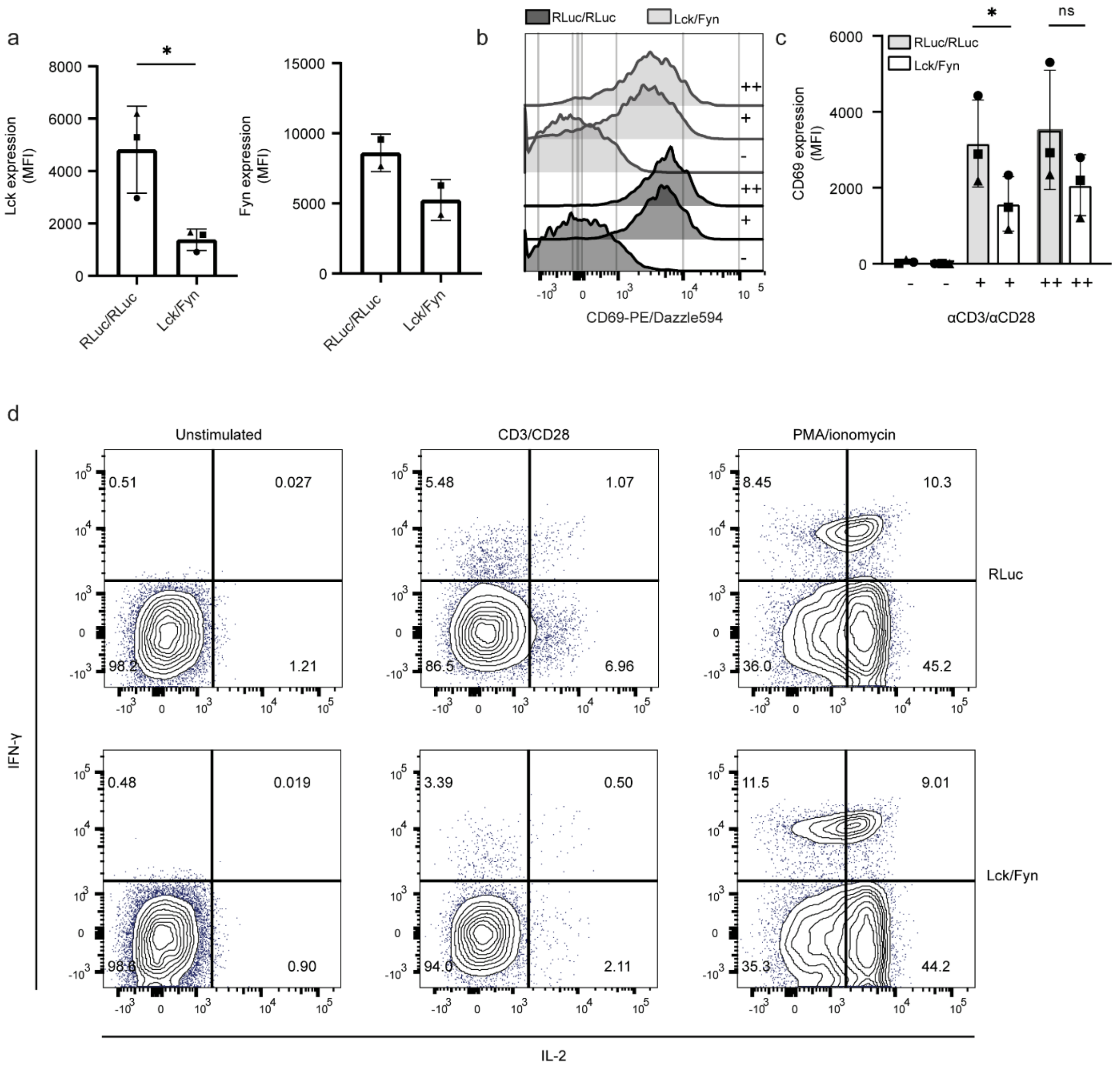

Supplemental Figure 4. Lck and Fyn double knockdown slightly impairs activation of human PBLs

(A) Aggregate data of total Lck and Fyn expression in human PBLs after transduction with microRNA vectors targeting Lck and Fyn. (B, C) Surface CD69 expression of human PBLs transduced with non-target microRNA vectors (dark grey) or double Lck and Fyn knockdown (light grey) left unstimulated (-), or upon TCR triggering by low (+) or high (++) dose anti-CD3/CD28. (A, C) Each symbol represents an individual donor (n=3) examined in a separate experiment, with bars depicting the mean and standard deviations. (D) Representative plots (n=3) of the effect of Lck and Fyn double knockdown on cytokine production by human PBLs. Significance was calculated with the paired student's *t*-test. *p<0.05.

Supplementary Table 1. Overview of the antibodies used in this study

Antibody	Clone	Manufacturer
Anti-mouse Ly6G/Ly6C	RB6-8C5	eBioscience
Anti-mouse CD90.2	30-H12	Biolegend
Anti-mouse IgG1	RMG1-1	Biolegend
Mouse IgG1, isotype ctrl	MOPC-21	Biolegend
Anti-mouse/human TCRζ	6B10.2	Biolegend
Anti-human CD4	RPA-T4	Biolegend
Anti-human CD8α	RPA-T8	Biolegend
Anti-human CD45	HI100	Biolegend
Anti-human CD45RA	HI100	Biolegend
Anti-human CD27	O323	Biolegend
Anti-human CD69	FN50	Biolegend
DYK/FLAG tag	L5	Biolegend
Anti-human CD3ε	UCHT1	Biolegend
Anti-human CD3ε	OKT3	Biolegend
Anti-human CD3ε	SK7	Biolegend
Anti-human CD3ε	HIT3a	Biolegend
Anti-human CD28	CD28.2	Biolegend
Anti-human TCRβ	IP26	Biolegend
Anti-human Lck	LCK-01	Biolegend
Anti-human ZAP70	1E7.2	Biolegend
Anti-human IL-2	MQ1-17H12	Biolegend
Anti-human IFN-γ	B27	Biolegend
Anti-human Actin (beta)	W16197A	Biolegend
Anti-human phospho-tyrosine	PY20	Biolegend
Anti-human Fyn	FYN-59	Biolegend
